# Multimodal Functional Network Connectivity: An EEG-fMRI Fusion in Network Space

**DOI:** 10.1371/journal.pone.0024642

**Published:** 2011-09-22

**Authors:** Xu Lei, Dirk Ostwald, Jiehui Hu, Chuan Qiu, Camillo Porcaro, Andrew P. Bagshaw, Dezhong Yao

**Affiliations:** 1 The Key Laboratory for NeuroInformation of Ministry of Education, School of Life Science and Technology, University of Electronic Science and Technology of China, Chengdu, China; 2 Key Laboratory of Cognition and Personality (Ministry of Education) and School of Psychology, Southwest University, Chongqing, China; 3 School of Psychology, University of Birmingham, Birmingham, United Kingdom; 4 Department of Neurology and Bermstein Center for Computational Neuroscience, Charité, Berlin, Germany; 5 Institute of Neuroscience, Medical School,Newcastle University, Newcastle, United Kingdom; Cuban Neuroscience Center, Cuba

## Abstract

EEG and fMRI recordings measure the functional activity of multiple coherent networks distributed in the cerebral cortex. Identifying network interaction from the complementary neuroelectric and hemodynamic signals may help to explain the complex relationships between different brain regions. In this paper, multimodal functional network connectivity (mFNC) is proposed for the fusion of EEG and fMRI in network space. First, functional networks (FNs) are extracted using spatial independent component analysis (ICA) in each modality separately. Then the interactions among FNs in each modality are explored by Granger causality analysis (GCA). Finally, fMRI FNs are matched to EEG FNs in the spatial domain using network-based source imaging (NESOI). Investigations of both synthetic and real data demonstrate that mFNC has the potential to reveal the underlying neural networks of each modality separately and in their combination. With mFNC, comprehensive relationships among FNs might be unveiled for the deep exploration of neural activities and metabolic responses in a specific task or neurological state.

## Introduction

Exploring long-range interactions of neuronal assemblies at different temporal and spatial scales is an important issue in human brain research. The concept of brain functional connectivity, defined as the statistical dependence between neuronal activities in distant regions, is central for the understanding of the organized behavior of cortical regions which constitute distributed functional networks (FNs) for cognitive and perceptive processing [1]. Activity in one neural system can directly or indirectly exert influence on another. This influence is modeled as effective connectivity in the brain, and has been extensively investigated with electroencephalography (EEG) and hemodynamic measurements [2], [3].

EEG and functional Magnetic Resonance Imaging (fMRI) recordings provide complementary information about brain activation and may help to explain the complex relationships among brain regions [4]. However, volume-conducted and convolved hemodynamic signals are spatially and temporally ‘mixed’ across the brain [5]. Independent component analysis (ICA) is a useful approach to decompose these mixed signals [6]. For fMRI, the temporal dynamics of independent components (ICs) have been further utilized to examine the functional interactions among different correlated brain networks [7]. Functional network connectivity (FNC) is a powerful approach to characterize the relationships between distributed brain networks, while functional connectivity (FC) focuses upon the relationships between voxels or regions. The nodes in FNC are defined as FNs that contain multiple brain regions. Previous studies used the lag between time courses of FNs to examine FNC differences between schizophrenic and healthy controls [7]. A recent extension using Granger causality analysis (GCA) provides a powerful way of studying the directional interactions among FNs [8].

To date, all FNC estimations have been computed from a single modality (i.e., fMRI). Based on high-resolution EEG, Babiloni and colleagues [9] employed fMRI as spatial priors for EEG source inversion. The time course utilized to infer causality, however, was still single modality EEG data, i.e., the cortical current density waveforms. A thorough investigation of the relationship among FNs needs to integrate the information from other modalities. A straightforward extension of fMRI FNC may cover the interaction among EEG FNs; meanwhile the FNs from different modalities can be matched using EEG-fMRI fusion [10]. Based on the assumption of linear neurovascular coupling, previous studies convolved EEG features with a standard hemodynamic response function (HRF) to model the time-series of fMRI signal [11]. In this fashion, the hemodynamic correlates of EEG rhythms [12], [13], [14] and adaptive modulations of event related responses were studied [15]. In a resting state data study, fMRI FNs were temporally correlated with power fluctuations in different bands from concurrently recorded EEG [16]. Recently we provided a source localization method based on multiple fMRI FNs: NEtwork-based SOurce Imaging (NESOI) [17], [18]. NESOI can reconstruct the neural source activation for each EEG IC, and more importantly, EEG and fMRI ICs can be matched in the spatial domain with the hyperparameters of NESOI.

In this study, we present a novel approach to estimate multimodal functional network connectivity (mFNC), a computational method that explores the direction of information flow among distributed brain networks gathered from simultaneous EEG and fMRI recordings. First, the functional brain networks from a single modality are extracted using spatial ICA. Then the directed interactions among FNs are explored using causality analysis. Finally, the fMRI FNs are matched to EEG FNs in EEG source inversion, which combines EEG-FNC and fMRI-FNC into an mFNC. MFNC has the potential to yield a holistic EEG-fMRI-based picture of the interactions among brain networks. In the current study, the performance of mFNC is demonstrated using simulated data and real EEG and fMRI signals collected during checkerboard stimulation.

### Theory

**Figure 1:**
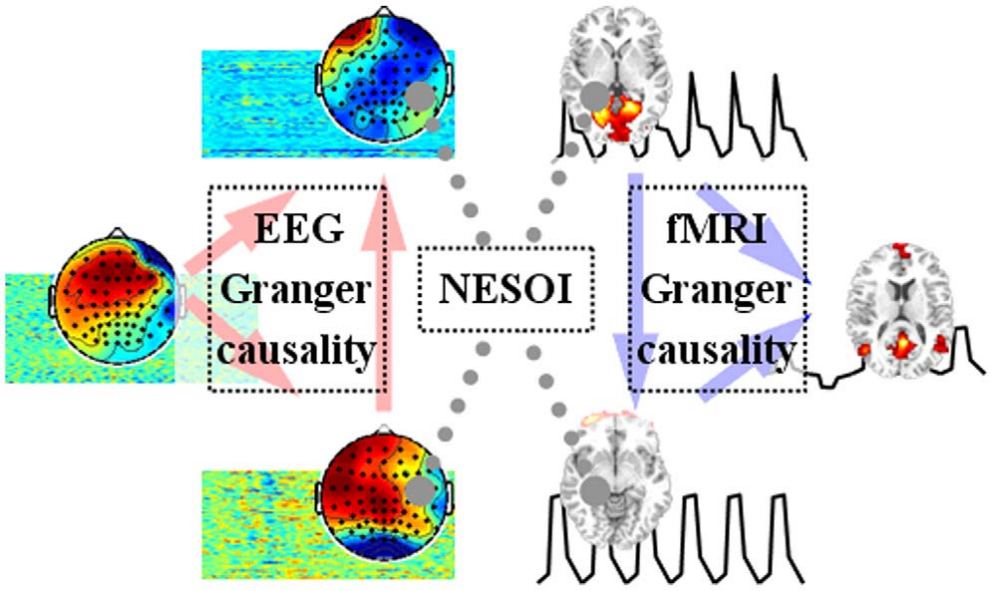
Illustration of the multimodal functional network connectivity. Functional networks (or Independent components) are identified using ICA from EEG and fMRI signal respectively. The time courses of EEG and fMRI networks are entered into Granger causality analysis to explore the interactions between networks, while their corresponding spatial patterns are matched by NESOI to combine EEG-FNC and fMRI-FNC into an mFNC. This yields a holistic expression of the interactive relationship between brain networks.

Multimodal functional network connectivity (mFNC) is a natural extension of fMRI FNC to cover the interaction among EEG FNs and to further explore the spatial matching between different modalities. The core procedures of mFNC are illustrated in Figure 1 and will be explained in detail in the following section.

In summary, mFNC first explores causal connectivity among brain networks with EEG and fMRI signals separately. To do so, EEG and fMRI data undergo modality-specific preprocessing i.e. spatial normalization of fMRI volumes and artifact removal of EEG (see Section “Data Preprocessing” for a detailed description in the real data test). Then, spatial ICA is performed on the EEG and fMRI data separately (the main points are summarized in Section “Extracting Functional Networks”). The time courses of EEG (or fMRI) components are employed to explore the networks' interactions (Section “Exploring Influences between Functional Networks”). Second, the spatial patterns of fMRI are linked to the topographies of EEG using NESOI, which stitches EEG-FNC and fMRI-FNC to construct mFNC (Section “Matching between Modalities”). The final networks of mFNC are analyzed with graph theory (Section “Graph Theoretical Analysis”). In our computer simulation, the code for data generation and visualization, and the simulated data used here are collected in a customized STEFF toolbox which is available from the authors upon request.

#### Extracting Functional Networks

Blind source decompositions are implemented to extract FNs in each modality. ICA is a generative “latent variable” model that describes how the observed data are generated by a process of mixing the underlying unknown sources [19]. The sources are assumed to be statistically independent and non-Gaussian. The results of the decomposition are *n* spatial ICs (topographies for EEG or spatial patterns for fMRI) and a mixing matrix consisting of the corresponding *n* time courses. This process condensed the whole brain activation into *n* components. The number of components is determined by the number of electrodes for EEG and the number of time points for fMRI. The common components across multiple implementations can be identified by cluster analysis [20]. The FastICA toolbox (http://www.cis.hut.fi/projects/ica/fastica/) was used for both EEG and fMRI ICA. After artifacts removal, the remaining FNs pave the way for NESOI and GCA.

#### Exploring Influences between Functional Networks

Granger causality analysis (GCA) estimates causality based on EEG and fMRI temporal information to explore directed influences between FNs. GCA implements a statistical interpretation of causality in which *S*_i_ “Granger causes” *S*_j_ if knowing the past value of *S*_i_ can help predict *S*_j_ better than knowing the past of *S*_j_ alone [21], [22]. The standard implementation of GCA is achieved via vector autoregressive (VAR) modeling, in which a set of time series are modeled as weighted sums of past values. Let *S*(*t*)  =  [*s*_1_(*t*), *s*_2_(*t*),...,*s*_k_(*t*)]*^T^* be a *k*-dimensional random process defined in a segment of windowed time series data, where *T* stands for matrix transposition. Assuming stationarity of the process *S*(*t*), one can describe *S*(*t*) by a *p*th-order autoregressive process:
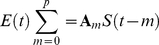
(1)where **A** *_0_* is the identity matrix and **A** *_m_* (*m* = 1, 2,..., *p*) are *k*×*k* coefficient matrices. *E*(*t*) is a *k*-dimensional, zero mean, uncorrelated noise vector. **A** *_m_* matrices can be estimated by the Levinson-Wiggins-Robinson (LWR) algorithm [23]. The covariance matrix of the noise (**Σ**) is estimated from the Yule-Walker equations of the model. The multivariate Bayesian Information Criterion (BIC) is calculated to determine the VAR model order, *p*.

Once **A** *_m_* and the covariance matrix of the noise (**Σ**) are estimated, the Granger causal influence from *S*_2_ to *S*_1_ can be inferred if knowing *S*_2_ reduces the variance in *S*_1_'s prediction error when all other variables *S*_3_ … *S*_n_ are also included in the regression model. To avoid excessive mathematical complexity we skip the derivation of GCA and refer to the literature [22], [24] for details. For event-related data, each trial is considered to be an independent realization of a single statistically stationary process, such that a single VAR model can be estimated based on the entire data set.

Having computed Granger causality magnitudes, statistical significance is established via an F-test on the null hypothesis that **A** *_m_*(*i*,*j*) is zero. These tests are corrected for multiple comparisons using the Bonferroni correction, in which the applied threshold is *p*_nom_/*n*(*n*-1), where *p*_nom_ is the nominal threshold (here we use 0.01), and *n* is the number of nodes. In the current study, Granger causality analysis was calculated using the GCCA toolbox (www.anilseth.com). Regarding FNs as nodes and influences as edges, both EEG-FNC and fMRI-FNC are constructed at this stage.

#### Matching between Modalities

**Figure 2:**
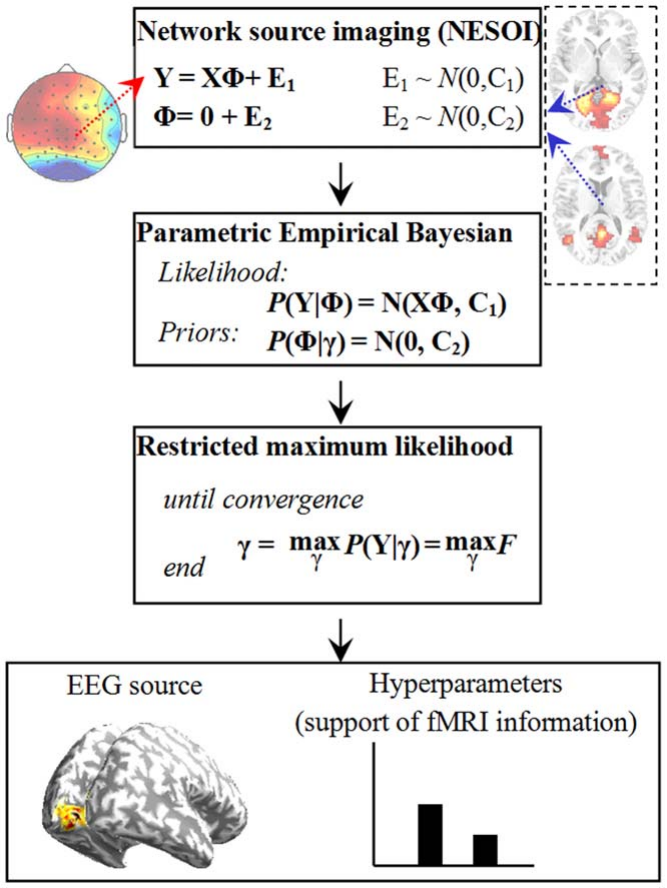
A schematic illustration of the architecture of NESOI and its computation scheme. NESOI employs multiple fMRI functional networks (fMRI ICs, top right) to reconstruct the source of each EEG functional network (EEG IC, top left). The Parametric Empirical Bayesian model is inverted by the Restricted Maximum Likelihood (ReML) algorithm. The products of ReML iteration are the conditional density of EEG source distribution and hyperparameters that quantify the matching between EEG and fMRI networks.

The fMRI FNs are matched to EEG FNs using network source imaging (NESOI), which implements Parametric Empirical Bayesian (PEB) models to find the EEG-fMRI common FNs. As illustrated in Figure 2, NESOI employs fMRI FNs (spatial patterns) as the covariance priors (**C**_2_) to reconstruct the neuroelectric source (*Φ*) of the EEG FN (topography: *Y*) [18], [25]. To guarantee that the covariance priors have a sufficient sampling of the source space, the subspace uncovered by fMRI FNs is added in as multiple sparse prior (MSP see [26]) in NESOI. Each prior (an fMRI FN or MSP) is assigned a non-negative hyperparameter that controls the relative contribution. After model inversion, the hyperparameters corresponding to the priors can identify whether an EEG FN can be considered as fMRI supported or unsupported component. The FNs are divided into three categories: fMRI-supported EEG FNs along with their corresponding fMRI FNs are the EEG-fMRI common FNs; the fMRI-unsupported EEG FNs are the EEG-specific FNs; the remaining fMRI ICs are the fMRI-specific FNs. The matched EEG-fMRI common FNs are assumed to represent the same neuronal populations, and are the key nodes in combining EEG-FNC and fMRI-FNC.

The PEB model in NESOI is inverted using the Restricted Maximum Likelihood (ReML) algorithm [27]. As illustrated in Figure 2 and the Text S1, the covariance at the second-level, **C**_2_, is determined by hyperparameters 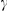, which are akin to the standard regularization parameters in ill-posed problems and need to be inferred from the observed data. The objective function maximized by ReML is identical to the variational free-energy [27]. In fact, the free-energy provides a tight lower bound on the model's log-evidence, 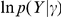, which increases with the accuracy of the model but decreases with the complexity [26]. ReML yields the conditional density of the source neuroelectric activity, and 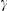 quantifies the support from the fMRI FNs to each EEG FN. In fact, the ReML iteration yields a parsimonious model, which makes EEG and fMRI FNs match sparsely. The implementation of ReML algorithm was performed with the free academic software package SPM (http://www.fil.ion.ucl.ac.uk/spm/), and hyperparameters were used to identify the EEG-fMRI common substrates. Further mathematical details of NESOI are given in Text S1.

#### Graph Theoretical Analysis

The interactions among FNs are further analyzed with graph theoretical analysis (GTA), which allows quantitative comparison of EEG-FNC and fMRI-FNC. The causal density and causal flow [28] are the central concerns of this work. Causal density refers to the total amount of causal interactivity sustained by a network. It is a useful measure of dynamical complexity because high causal density reflects integration and differentiation in network dynamics [28]. In this case, nodes (functional brain networks) are both globally coordinated in their activity (useful for predicting each other's activity) and dynamically distinct (so that different elements contribute in different ways to these predictions). Causal flow refers to the difference between its out-degree (number of outgoing connections) and its in-degree (number of incoming connections). Causal flow can identify nodes that have distinctive causal effects on network dynamics: a node with a highly positive flow is a causal ‘source’; a node with a highly negative flow is a causal ‘sink’.

### Simulation

**Figure 3:**
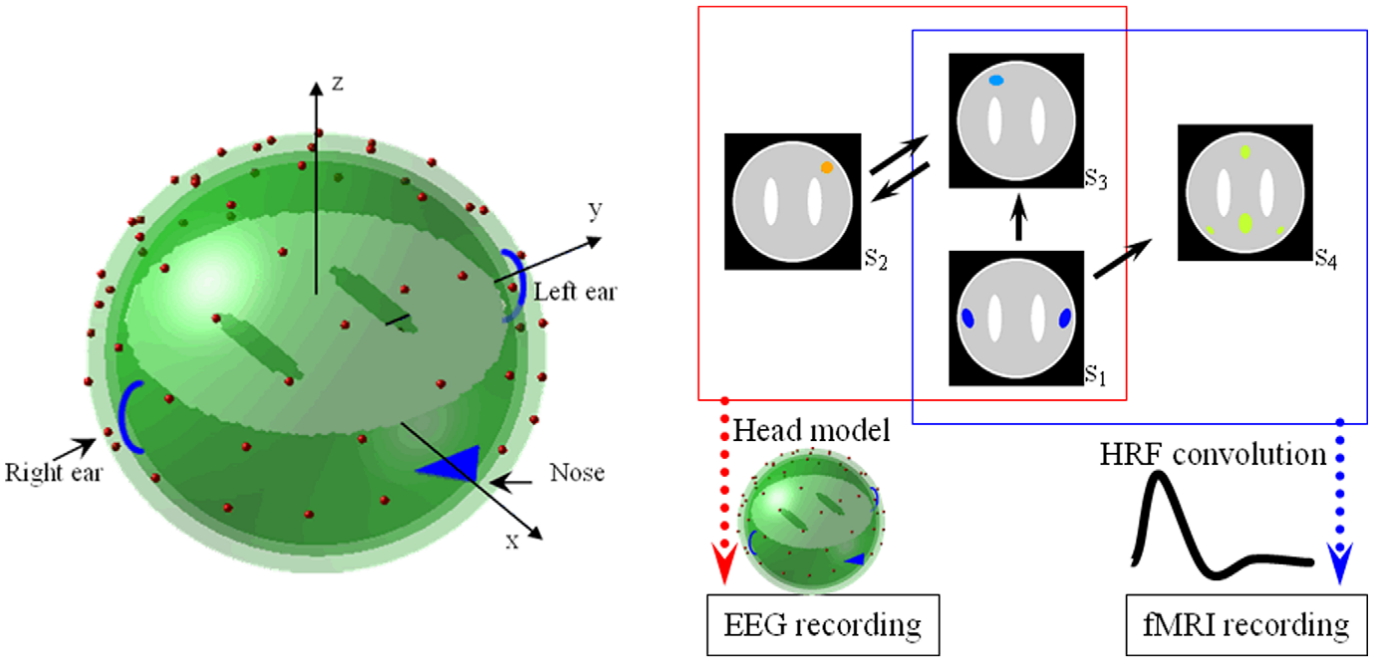
Head model and the construction of synthetic data. (a) Head model: 2452 voxels within a concentric three-sphere head model with 64 electrodes on the upper surface. The two holes in source slice are ‘white matter.’ (b) A schematic illustration of the procedure to generate simulated time series. The spatial profile of each source is drawn with different color and the background is shown in gray and white. EEG signals are recorded from S_1_, S_2_ and S_3_ (red-bordered areas), which generate scalp potentials through the head model. S_1_, S_3_ and S_4_ (blue-bordered areas) are filtered by convolution with a gamma function to yield fMRI recording. S_2_ and S_4_ are assumed to be fMRI and EEG blind respectively.

To illustrate the performance of mFNC in exploring the underlying causal relationship, a disc with 2452 voxels/dipoles is employed to generate the synthetic data. In Figure 3(a), the disc was wrapped with a concentric three-sphere EEG head model with 64 electrodes placed on the upper hemisphere. The two holes in the disc represent areas of ‘white matter’. In the fMRI scanner, one slice with Z-axis coordinate of 18 mm and field of view (FOV) of 200×200 mm^2^ covers this disc, and generates a two-dimensional fMRI spatial map of 70×70 voxels. In Figure 3(b), the spatial profiles of four sources are drawn with different colors on the disc. Each source is pretended to represent a possible neurophysiological structure: ‘auditory cortex’ *S*_1_, ‘right cognition area’ *S*_2_, ‘left cognition area’ *S*_3_ and ‘default mode networks’ *S*_4_.

The following autoregressive process is used to simulate the neuronal interactions among the four sources:
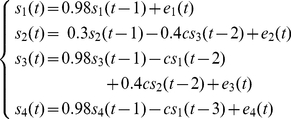
(2)where 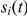 and 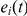 is one channel of random process S(*t*) and white noise **E**(*t*) in Equation (1). The parameter *c* represents the coupling strength, ranging from 0.1, weak coupling, to 0.7, strong coupling. **Σ** is the covariance matrix of the noise **E**(*t*) and is set to the identity matrix. The number of simulated data points is 18,000 (360 s × sample rate 50 Hz) for each source of the VAR-process. Initial 1,000 points are additionally simulated and later discarded to allow the system to enter into a steady state. Figure 3 summarizes causal relationships between sources, where a solid arrow from *S*_j_ to *S*_i_ is drawn if *S*_j_ causes *S*_i_.

To mimic spatial decoupling between EEG and fMRI sources, *S*_2_ and *S*_4_ are assumed to be EEG and fMRI modality-specific sources, respectively. For the EEG recording, the neural time courses of *S*_1_, *S*_2_, and *S*_3_ are divided equally into 40 segments to simulate 40 trials (data points: 450 = 18,000/40). The first 55 time samples of each trial are used to simulate 1,100 ms of EEG recording (sampling interval 20 ms). After normalizing the three sources to zero mean and unit standard deviation, white Gaussian noise was added according to a signal-to-noise ratio (SNR) 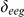 for each source, representing noise in the neuroelectric response. SNR is defined as the ratio between signal and noise variances. The scalp potential distribution is generated by computing the forward problem for the three sources simultaneously [29]. After renormalizing the scalp signals, another Gaussian noise with SNR of 1 was added to represent measurement error and noise in the scalp data acquisition. The electrodes from the experiment collecting the real data (see Section “Experiment and Data Acquisition”) were registered to the scalp surface, and the lead-field matrix (**X**) was calculated analytically based on a concentric three-sphere head model [30].

Based on a previous study [31], fMRI recording is approximated with a low-pass filtered and sub-sampled version of the above neural time courses. The signals of *S*_1_, *S*_3_ and *S*_4_ are individually filtered by convolution with a linear model of a gamma HRF [32]. After individually normalizing the signals to zero mean and unit variance, white Gaussian noise was added to represent noise in the hemodynamic signals with SNR 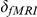. Subsequently, these simulated BOLD signals were sampled every 75 time-steps to simulate signal acquisition by the scanner with a whole volume repeat time (TR) of 1.5 s (75 × the sampling interval 20 ms), yielding signals with 240 time samples. After renormalizing, another Gaussian noise was added to represent measurement noise with SNR 0.2 in the fMRI data acquisition [31].

It is worth mentioning that the current simulation introduces noises at three independent stages: the 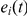 in Equation (2) that drives the dynamic system to generate neuronal interactions, the physiological noise added in neuroelectric (for EEG) and hemodynamics (for fMRI) responses and the noise at the level of sampling to mimic measurement noise. Other parameters are similar to our previous study [17] and are listed in Table 1.

**Table 1:** Parameters in simulation.

EEG	fMRI
Sources: *S*_1_, *S*_2_, *S*_3_	Sources: *S*_1_, *S*_3_, *S*_4_
Sampling rate: 50 Hz	Repeat time: TR = 1.5 s
Number of time samples: 55×40 (trials)	Number of time samples: 240
SNR of physiological noise: 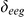	SNR of physiological noise: 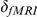
SNR of measurement noise: 1	SNR of measurement noise: 0.2
Number of electrodes: *e* = 62	Size of simulated HRF: *l* = 13
Lead-field matrix **X**: 3 spheres head model with analytic solution Sphere radii: [0.87 0.89 1]	HRF function: Gamma function70×70 voxels Field of view (FOV): 200×200 mm^2^
Signal recording length: 360 s	
Number of dipoles/voxels: *d* = 2452	
Number of dipoles per source (S_1_-S_4_): [90 30 32 100]	

#### Procedure of mFNC

**Figure 4:**
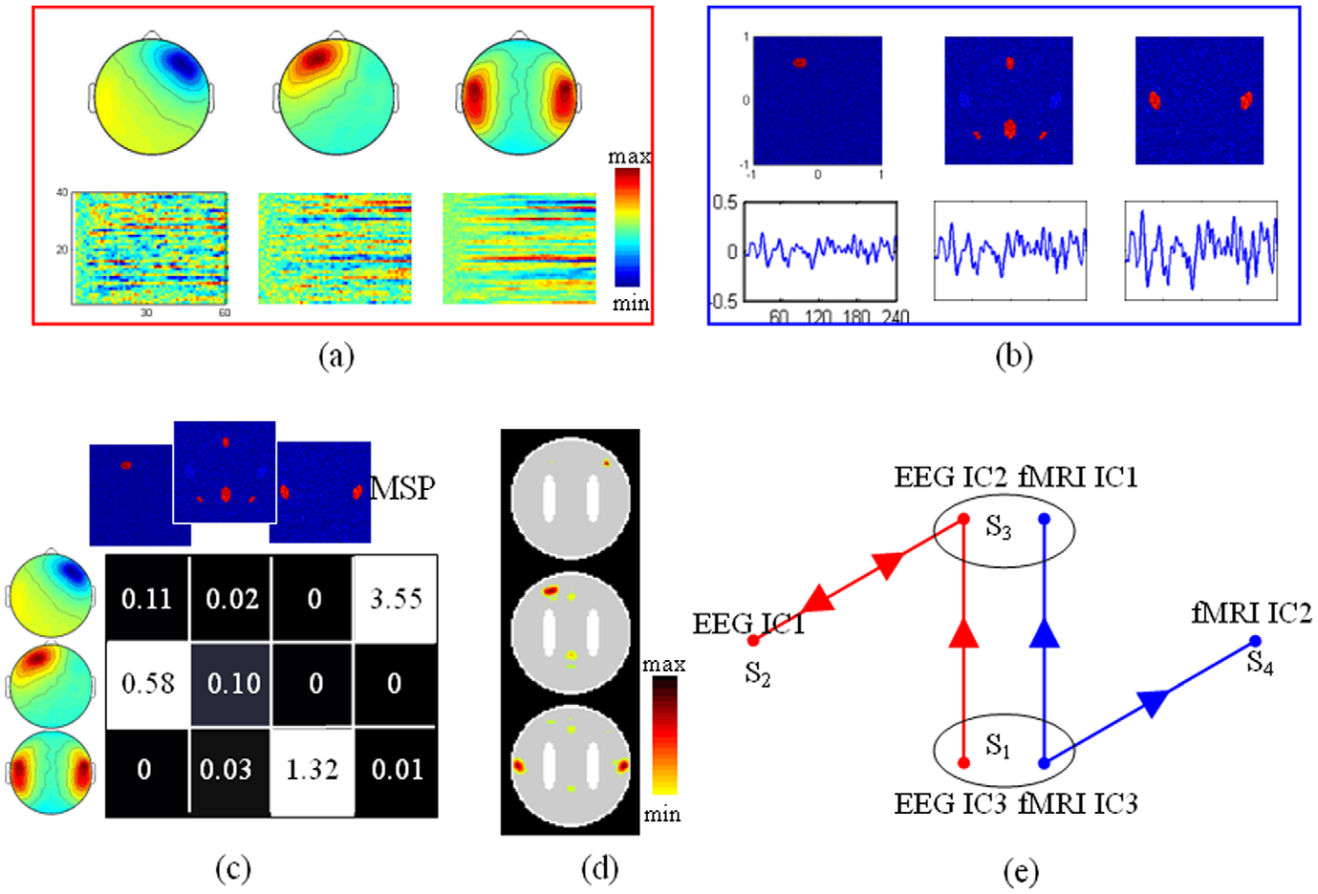
An illustration of the intermediate results of mFNC in simulation. (a) scalp potential distribution (1^st^ row) and single trial images (2^nd^ row) of EEG components, which are extracted by employing spatial ICA; (b) spatial distribution (1^st^ row) and the corresponding BOLD signals (2^nd^ row) of fMRI components obtained using spatial ICA; (c) the hyperparameters quantify the support from the fMRI spatial patterns (the top row) for each EEG component (the left column); (d) source localization results corresponding to the extracted EEG ICs. The maps are shown with a threshold of 1% quantile of the spatial distribution; (e) Granger causality estimated from EEG and fMRI signals, S_1_ and S_3_ are the EEG-fMRI common sources. Red arrows depict connections for EEG and blue arrows for fMRI. There is a bidirectional link between EEG IC1 and IC2.

An implementation of mFNC is shown in Figure 4 with parameters of *c* = 0.5, 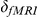 = 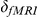 = 5. In the FNs extracting stage, FastICA iteration usually terminated after extracting three components, because the fixed point algorithm did not converge in further calculating. The common FNs across our 20 implementations were identified by cluster analysis, and the final three clustering centers were used as spatial IC for each modality. Figure 4(a) and 4(b) illustrate the FNs used for the following procedures of NESOI and GCA.

NESOI reconstructed the source activity of EEG FNs in Figure 4(d), and the estimated hyperparameters are illustrated in Figure 4(c). Each row shows the relative contributions of fMRI FNs (in the top row) to the EEG FNs (in the left column). The grayscale of each row is normalized to emphasize the maximum with white color. Obviously hyperparameters indicate the accurate relationship between modalities: EEG IC1 is an EEG-specific FN that MSP has the largest value, EEG IC2 is matched to fMRI IC1, EEG IC3 is matched to fMRI IC3. As both EEG IC2 and fMRI IC1 have the largest activation in *S*_3_, they all construct the common source *S*_3_. With similar property, EEG IC3 and fMRI IC3 construct the common source *S*_1_. Model special component EEG IC1 and fMRI IC2 are assigned to source *S*_2_ and *S*_4_ respectively. Consequently, 4 sources are constructed in the mFNC and the directed edge between them will be explored with Granger causality. Another product of NESOI is the neuroelectric activity imaging in Figure 4(d), where all the EEG visible activated areas in Figure 3 are well reconstructed. However, the sources of EEG IC1 are more local than the simulated one in Figure 3. The sources of EEG IC2 and EEG IC3 are contaminated by inaccurate priors from MSPs and fMRI IC2, as there are some small clusters around the central areas.

Time courses of EEG and fMRI FNs are used to estimate the Granger causality. The VAR model order estimated by BIC is two for EEG data and one for fMRI data. The resulting Granger causalities among FNs are illustrated in Figure 4(e). Edges explored from EEG and fMRI are shown with different colors. The estimated edges from both modalities indicate that S_3_ receives information from S_1_. In contrast, FNCs derived from single modality cannot identify the causal granger relationship between these sources and the other blind (modality-specific) sources, such as S_2_ for fMRI and S_4_ for EEG. Considering the EEG IC1 and fMRI IC2, the directed influences illustrated in Figure 4(e) together correctly show the entire networks simulated in Figure 3.

#### Robustness of mFNC

The reconstruction of mFNC contains multiple stages: extracting FNs, exploring the influences among FNs and matching between modalities. Previous studies have statistically investigated the performance of ICA decomposition [6], [20] and EEG source imaging [18]. VAR model and Granger causality have been proven to be capable of exploring the patterns of neural interactions based on neuroelectric signals [22]. Granger causality has also been applied to fMRI signals even if the temporal sampling of BOLD responses is larger than the time scale of the influence [31]. In this simulation, instead of a detailed consideration of noise effect and coupling strength on each stage, we investigate the overall effect that to what extent mFNC is capable of detecting directed interactions among neuronal populations.

**Figure 5:**
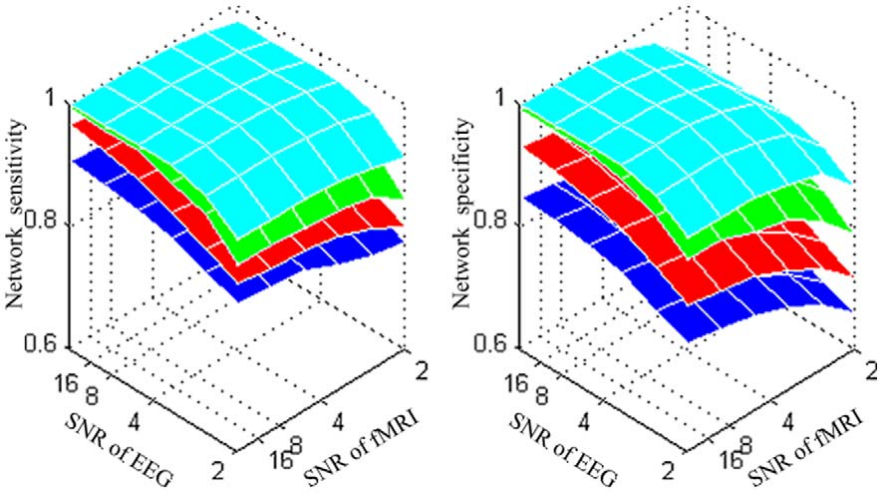
Mean value of sensitivity (proportion of true positive) and specificity (proportion of true negative) over 256 simulations. Four surfaces show the performance for *c* = 0.1 (blue surface), *c* = 0.3 (red surface), *c* = 0.5 (grey surface), and *c* = 0.7 (cyan surface), as a function of different noise levels of EEG 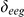 and fMRI 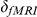.

Systematic combinations of different levels of parameters include: the strength of influence (*c* = 0.1, 0.3, 0.5, 0.7), the SNR of EEG (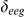  = ∞, 10, 5, 3.3, 2.5, 2) and fMRI (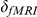  =  ∞, 10, 5, 3.3, 2.5, 2). For each of the 144 (4×6×6) possible combinations of these parameters, a set of 256 simulations was performed and mFNC was reconstructed on the simulated signals. The performance of the reconstruction was evaluated using rigorous criteria. Though the final evaluation is for the reconstructed interactions, here we follow a two-step procedure: first, based on NESOI, the FNs of EEG and fMRI are classified into different groups (for example the four groups illustrated in Figure 4(e)) with two of them being matched FNs (*S*_1_ and *S*_3_); second, only the correct Granger results among the correct groups are taken as the correct results. We evaluated the reconstructed interactions among sources with sensitivity (proportion of true positive) and specificity (proportion of true negative). The mean values of 256 implementations are given in Figure 5, which plots sensitivity and specificity as a function of a range of values for *c*, 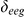 and 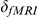.

The four surfaces show the overall influences of the parameter to ICA decomposition, EEG source imaging and causality analysis. Three main observations can be made. First, an increase in the coupling strength at given levels of SNR leads to a steady increase in the sensitivity and specificity. Second, increasing SNR when keeping coupling strength also increases the influence measure. The conjoint effects of EEG and fMRI noise seem to be roughly additive. Third, sensitivity mainly depends on EEG noise while specificity depends on both noises. Further checking the details of the EEG FNs reveals that the decrease of EEG SNR will aggravate ICA decomposition, and this leads to failure in reconstructing of the sources of mFNC, and further the edges between them. In contrast, fMRI noise has little influence on detection of interaction. Previous literature indicated that the sensitivity decreases rapidly with increasing sampling interval and decreasing influence delay [31]. In our simulation, the main effect of fMRI noise is that the specificity decreases with increasing fMRI noise. Overall, these simulations suggest that the proposed method is able to detect influence between neuronal populations in simultaneous EEG-fMRI recording. However, the sensitivity to such interactions decreases rapidly with decreasing SNR and coupling strength.

### Experiment and Data Acquisition

As part of a study to investigate the link between EEG and fMRI at the level of single trials [33], a twenty-eight-year-old right-handed male was paid for his participation. Written informed consent was obtained and the protocol was approved by the Research Ethics Board of the Birmingham University Imaging Centre. All the EEG and fMRI recoding experiments were conducted according to the principles expressed in the Declaration of Helsinki. Individual trials of the experiment consisted of a single presentation of a hemifield checkerboard stimulus for 1 s with phase reversal after 500 ms followed by a fixation period which was uniformly sampled from 16.5 to 21 s, discretized to 1.5 s. Individual runs consisted of 17 trials per contrast with fixation periods at the beginning and at the end, amounting to a total session length of 11 min (441 volumes×1.5 s). Contrasts were randomized and five of these runs were acquired, resulting in 85 trials per contrast. In the current study, only trials of high contrast level (c_Michelson_ = 1) were used for subsequent data analyses. A simple fixation task was performed to maintain the observer's attention: on a random selection of half of the trials of a given session, the fixation cross changed from a plus sign to an X during the fixation period at random time points, discretely (1.5 s) and uniformly sampled from the interval of 4.5–16.5 s after stimulus onset. The observer's task was to report the change in fixation by a button press using the index finger of the right hand. Hit rate and number of false alarms were presented to the observer at the end of each session. Stimuli were presented and behavioural data were collected using Psychotoolbox3 for Matlab (The Mathworks, Natick, MA). The timing of stimulus presentation was controlled by the MRI scanner volume trigger.

EEG data were recorded using a 64-channel MR compatible EEG system (BrainAmp MR Plus, Brain Products, Munich, Germany). The EEG cap consisted of 62 scalp electrodes distributed according to the 10–20 system and two additional electrodes, one of which was attached approximately 2 cm below the left collarbone to record the ECG, while the other was attached below the left eye for measurement of the electro-oculogram. Data were sampled at 5000 Hz. fMRI data were recorded using a 3-T Philips Achieva MRI scanner. EPI data (gradient echo pulse sequence) were acquired from 441 volumes and 20 slices (2.5×2.5×3 mm resolution, TR = 1500 ms, TE = 35 ms, SENSE factor = 2, flip angle = 80°), providing approximately half brain coverage in the dorsal-ventral direction. Slices were oriented parallel to the AC-PC axis of the observer's brain and positioned to cover the entire occipital cortex.

## Results

### Data Preprocessing

For EEG measurement, the gradient artifacts were removed using the average artifact subtraction approach. Ballistocardiogram artifact correction was performed using the Optimal Basis Set method [34]. The data were low-pass filtered at 25 Hz and down-sampled to 50 Hz, then concatenated. The preprocessed EEG data were further re-referenced to the average [30] and baseline corrected. For each trial, signal between -100 ms and 1000 ms related to the stimulus onset were preserved for further analysis.

For fMRI measurement, SPM8 was used for pre-processing. Functional image time series data were first corrected for differences in slice acquisition times, then warped into standard Talairach anatomical space, and smoothed with an isotropic 8-mm full-width-at-half -maximum Gaussian kernel. The data were then concatenated and 13 peri-stimulus time points (18 s) were preserved from each trial for further analysis.

### Extracting EEG Functional Networks

ICA linearly decomposed EEG data into 61 maximally independent components. We implemented FastICA for 20 times with random initialization. To determine which components were common across implementations, we performed cluster analysis on the component maps [20] and eleven common components were selected according to the resulted clustering centers. Three neurophysiologically implausible patterns were removed due to their multi-polar structure in the scalp potential configuration (see Figure S1). Eight FNs were selected for further investigation.

**Figure 6:**
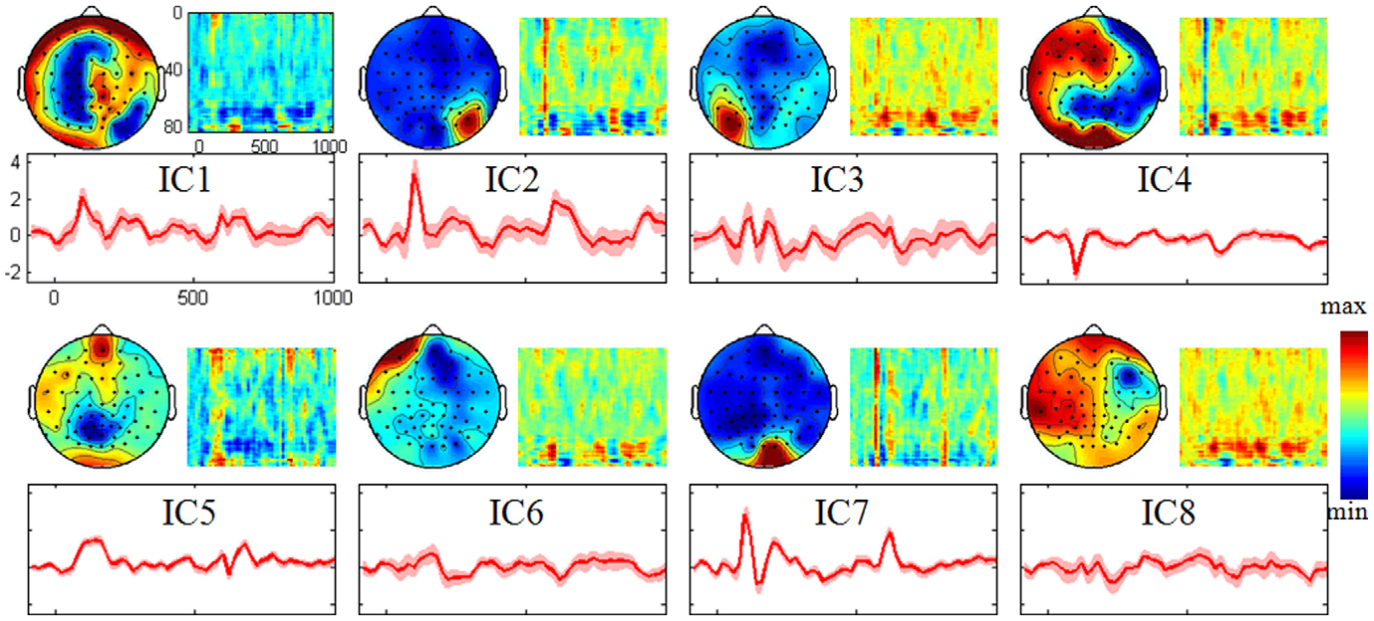
Visualization of the eight functional networks of EEG in real data test. For each network, three features are shown: scalp topography (top left insert); EEG single trial ERP image (top right insert, trials are smoothed by a 10-trials moving average for visualization); and the average EEG signal change (bottom insert. Red lines  =  ERP; semitransparent lines  =  standard error). Axes are the same for all features and are shown on subplot of IC1. IC2 and IC7 are stimulus sensitive functional networks, as their ERPs show a strong activity between 100 and 600 ms. The stimulus onset was at 0 ms, the stimulus reversal occurred at 500 ms.

Three primary features are presented in Figure 6 for each FN. The scalp topography (top left insert) corresponds to the decomposed spatial IC, whose time course is shown with a two-dimensional representation of ERP images (top right insert, trials are smoothed by a 10-trials moving average for visualization). The thin color-coded horizontal bars represent a single trial. The bottom insert is the average ERP during the visual tasks, where standard error is labeled with the semitransparent line.

### Extracting fMRI Functional Networks

The number of ICs in our fMRI data was determined by the number of time points. We implemented FastICA with random initialization and the common components across implementations are identified by cluster analysis [20]. The selected sixteen common components were further inspected to determine which fMRI components were likely to be of neurophysiological origin. We detected seven components that appeared to be associated with artifacts, such as head motion, cerebrospinal fluid, large vessels and dispersion of clusters (see Figure S2). Nine FNs were selected for further investigation.

**Figure 7:**
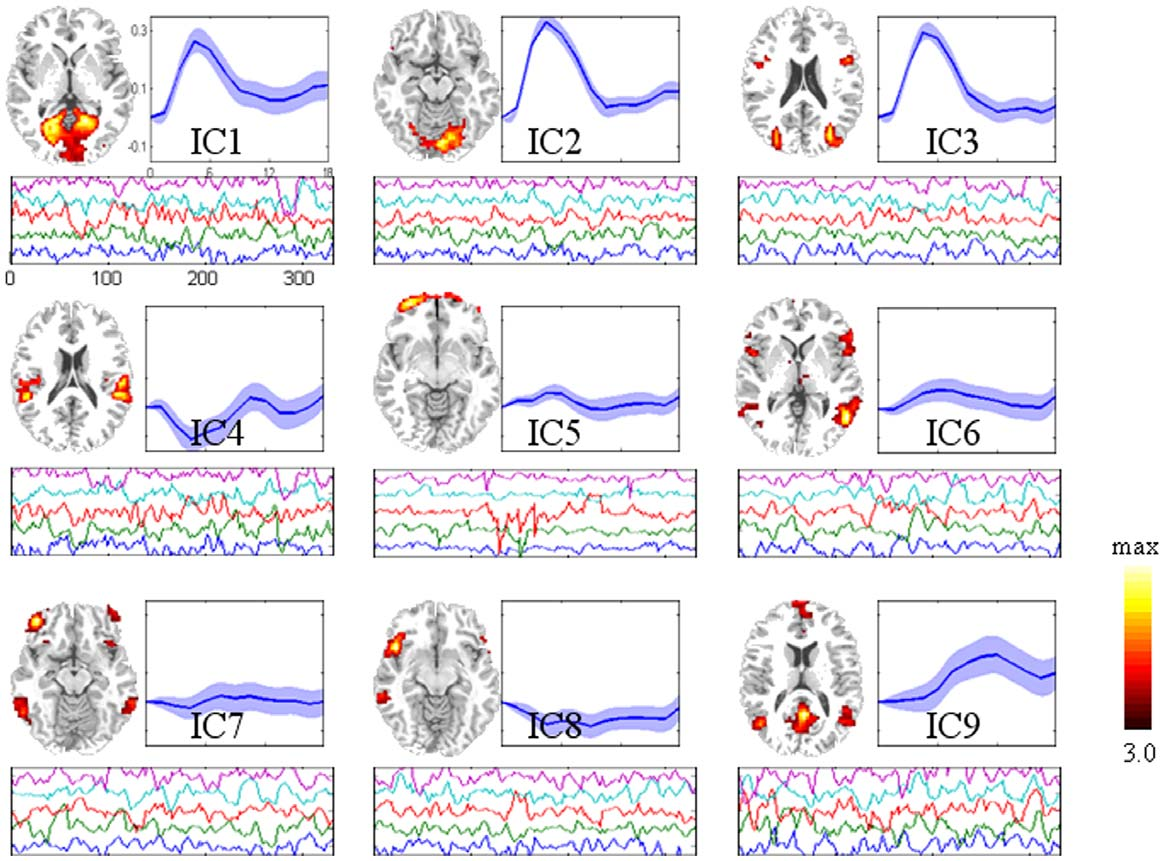
Visualization of the nine functional networks of fMRI in real data test. Three features are shown for each network: fMRI spatial distribution in an axial view (top left insert); the average BOLD signal change (top right insert. Blue lines  =  mean value; semitransparent lines  =  standard error); the corresponding BOLD time course in each run, five runs are colored with different colors (bottom insert). Axes are the same for all features and are shown on IC1. IC1, IC2 and IC3 are stimulus sensitive functional networks, as their average BOLD signals have strong activations about five seconds after the stimulated onset.

Three primary features are presented in Figure 7 for each component. The spatial distribution in an axial view corresponds to one slice of the fMRI FN. The intensities of each map were transformed to z scores. Voxels with absolute z scores higher than three were considered as activated and other detailed information (active regions, Brodmann's area (BA) and the corresponding network) are listed in Table 2. The corresponding time course is shown in the bottom insert, with five runs separately illustrated with different colors. The top right insert is the average BOLD signal for each FN.

**Table 2:** The active regions and corresponding networks of the fMRI independent components in real data test.

fMRI IC	Region	Brodmann area	Networks
**1**	Primary and visual association cortex	17, 18, 19	Association Visual Network
**2**	Primary visual cortex and a small area of visual association cortex	17, 18	Primary Visual Network
**3**	Visual association cortex and part of Broca's area	19, 44	Peristriate Visual Network
**4**	Primary and auditory association cortex	41, 42	Auditory Network
**5**	Orbitofrontal area, inferior and dorsolateral and prefrontal cortex	11, 47, 46	Prefrontal Network
**6**	Occipitotemporal areas	37	Visual-temporal Network
**7**	Inferior temporal and prefrontal gyrus	47, 20	Ventral temporal Network
**8**	Temporopolar areas	38	Temporopolar Network
**9**	Prefrontal area, posterior cingulate regions, the inferior temporal gyrus and angular gyrus	11, 23, 37, 39	Default Mode Network

### Matching between Modalities

**Figure 8:**
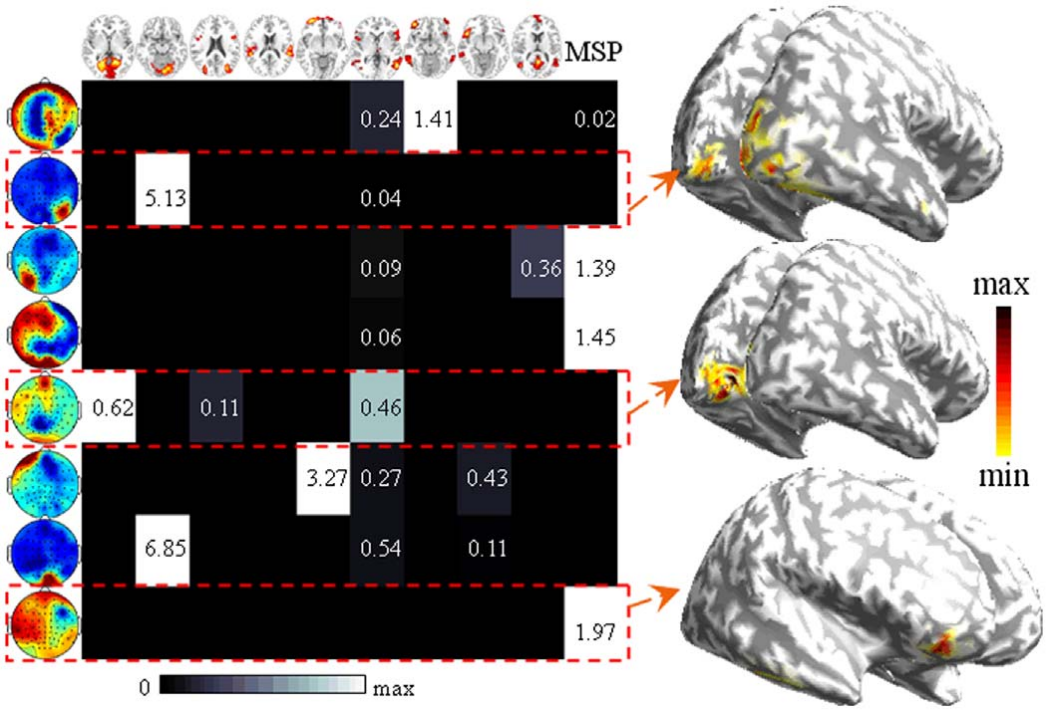
The results of NESOI in real data test. (a) The hyperparameters quantify the support from fMRI FNs (the topmost panel) to each EEG FN (the leftmost panel). The hyperparameters of FN IC2, 5 and 8 are highlighted with red dashed lines. (b) Three EEG source distribution displayed on the inflated cortex for EEG IC2, 5 and 8. The maps are shown with a threshold of 1% quantile of the spatial distribution.

Based on the FNs of the above processes, NESOI fuses EEG and fMRI FNs in the spatial domain for combining the EEG-FNC and fMRI-FNC into an mFNC in the following section. EEG source distribution and its corresponding hyperparameters are illustrated in Figure 8. In Figure 8(a), each row shows the relative contributions of the fMRI FNs in the topmost row to an EEG topography in the leftmost column. The grayscale of each row is normalized to emphasize the maximum with white color. The sources reconstructed from EEG ICs 2, 5 and 8 are displayed on the inflated cortex in Figure 8(b).

Obviously, only a few fMRI FNs are helpful for EEG source reconstruction. A straightforward consequence is that the relationship between EEG and fMRI is a sparse matching. EEG IC1 is matched to fMRI IC7 and they have some similar activations in the frontal areas. EEG IC2 is supported by fMRI IC2, which reflects the neural activity in primary visual cortex and a small area of visual association cortex. EEG IC5, 6 and 7 correspond to fMRI IC1, 5 and 2, respectively. FMRI IC3 has a task-related activation after the stimulus onset, but it does not support any EEG IC. This FN reveals that an fMRI visible source is invisible for EEG.

Another interesting result is the estimated hyperparameters of EEG IC3, 4 and 8, each having the largest value for MSP priors sampled sparsely from an EEG specific subspace [18], [26]. They will be referred as EEG-specific FNs in the following investigation. The brain region, Brodmann's area and MNI coordinates corresponding to the largest active position of each EEG FN after source reconstruction are listed in Table 3.

**Table 3:** Summary of source imaging results of NESOI in real data test.

EEG IC	Largest active position	Brodmann area	MNI coordinates	Matched fMRI IC
**1**	Superior Frontal Gyrus	11	[-27.28 61.68 -9.36]	fMRI IC7
**2**	Lingual Gyrus	18	[3.43 -74.84 -1.55]	fMRI IC2
**3**	Fusiform Gyrus	37	[-55.27 -49.06 -19.30]	N/A
**4**	Inferior Occipital Gyrus	17	[22.99 -95.58 -15.84]	N/A
**5**	Lingual Gyrus	18	[24.95 -76.70 -13.28]	fMRI IC1
**6**	Middle Temporal Gyrus	37	[52.25 -36.36 -15.00]	fMRI IC5
**7**	Lingual Gyrus	19	[-26.32 -66.33 3.35]	fMRI IC2
**8**	Middle Frontal Gyrus	47	[38.00 42.90 -11.85]	N/A

### Exploring Influences between Functional Networks

**Figure 9:**
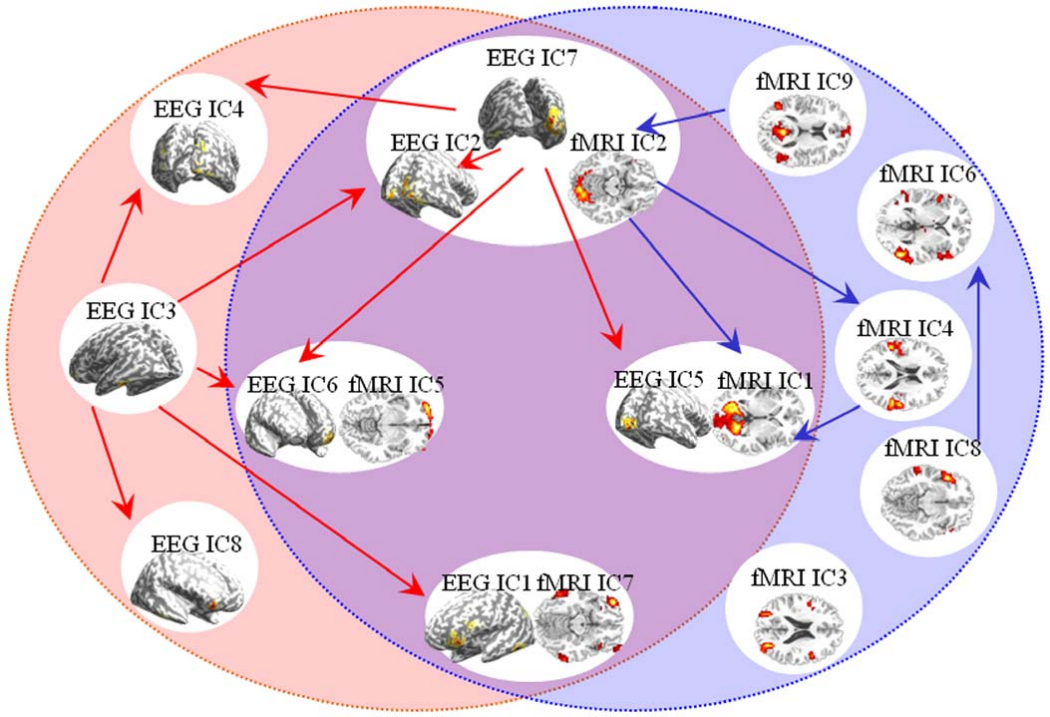
Multimodal functional network connectivity estimated from Granger causality analysis and NESOI in real data test. The left red and right blue ovals identify the functional networks of EEG and fMRI respectively. The middle intersection area of the red and blue ovals defines the “common substrate” of neuronal activity in the two modalities. Each small white oval represent the matched functional networks. Here the NESOI results on the inflated cortex are adopted as the substitute of the scalp EEG pattern. fMRI FNs are displayed on one slice of the anatomical image respectively. Red arrows depict connections for EEG and blue arrows for fMRI.

According to Figure 8 and Table 3, three categories of FNs emerged: EEG-specific FNs (red oval in Figure 9), fMRI-specific FNs (blue oval in Figure 9), and EEG-fMRI common FNs (the brown intersection area). The matched FNs estimated by NESOI are illustrated in a white oval. A Granger casual connectivity network was constructed with time courses of EEG and fMRI FNs separately. The VAR model order estimated by BIC is three for EEG and one for fMRI FNs. The edges with statistical significance values are labeled with red arrows when they are estimated from EEG signal, and are labeled with blue arrows when from fMRI. And all information in Figure 9 illustrates the mFNC derived from the real data.

For the EEG-fMRI common FNs (in the intersection area of the large red and blue ovals), the links between components are slightly different in the neuroelectric and hemodynamic domains. Only one edge is identified from the fMRI information. The fMRI IC2 is subdivided into two sub FNs for EEG: EEG IC2 and IC7 (in NESOI, fMRI IC2 supports both EEG IC2 and IC7, see Figure 8). Furthermore, EEG sub FNs revealed more information about signal flow: EEG IC7 was found to Granger-cause EEG IC2. Information flow from EEG IC7 to EEG IC6 was also reconstructed, which indicates that the frontal areas are activated by central occipital areas.

Generally speaking, both EEG and fMRI evidences are in accordance with the information processing of visual hierarchy. The occipital primary visual cortex (EEG IC3, IC7 and fMRI IC2) is the first stage of visual processing. Stimulation in the left visual field yields the activation in the right occipital cortices (see fMRI IC2), and this is consistent with the retinotopic organization of early visual cortex. Then, visual information flows further to the visual association cortex (EEG IC5 and fMRI IC1). A wide variety of visual primitives are processed here [35] and extended bilateral activation can be found in fMRI IC1.

EEG and fMRI specific information also reveal some interesting links between distributed brain regions. EEG IC3 has the largest positive activation in the left occipital region, which may be the projections of the opposite visual areas (such as EEG IC7). In the estimated networks, many edges are sent out from EEG IC3. Following the visual pathways, the activations in the left (EEG IC3) and right (EEG IC7) occipital regions are translated to bilateral and frontal areas (EEG IC4 and IC6). For fMRI-specific FNs, a link is reconstructed from DMN (fMRI IC9) to primary visual network (fMRI IC2). Previous studies have identified the DMN as the task-negative network, which is active when the individual is not focused on the outside world and the brain is at wakeful rest. The link between DMN and primary visual network may be the result of short rest between the simple visual stimulus tasks. An arrow from fMRI IC8 to IC6 is reconstructed. However, considering the overlapped temporopolar and occipitotemporal regions in left hemisphere, this link may be the result of over-separation of the ICA algorithm.

### Graph Theoretical Analysis

Based on the Granger causality network estimated by GCA, graph features of the networks were quantitatively characterized by GTA.

**Figure 10:**
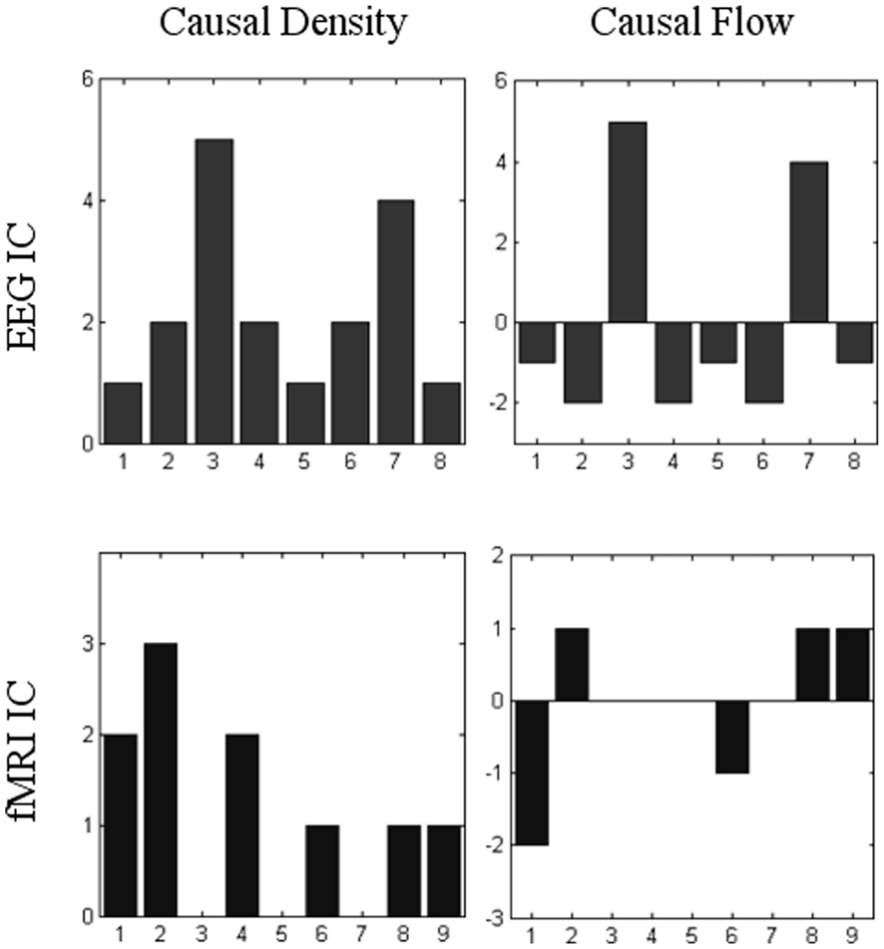
Causal density and causal flow of each EEG and fMRI functional networks in real data test. X-axis represents the index of IC.

As illustrated in Figure 10, causal densities and flows were calculated in each modality. There are nine links between the eight EEG FNs. In these FNs, EEG IC3 is an active node, with five edges flowing to other FNs. Another high causal density note is EEG IC7, which is matched to the high causal density node of fMRI IC2. From a causal flow perspective, EEG IC3 and EEG IC7 are the main causal ‘sources’ and they affect other FNs. In contrast, EEG IC2, 4 and 6 are the main causal ‘sinks’ with higher in-degree than out-degree, indicating that they are Granger-caused by the other nodes.

The connections between the nine fMRI FNs decreased to five. In these FNs, fMRI IC2 communicates widely and it has three edges connected to other nodes. For causal flow, fMRI IC2, 8 and 9 are the main causal ‘sources’, in which the difference between out-degree and in-degree is 1, and fMRI IC1 is the main ‘sink’, in which the difference between out-degree and in-degree is -2 (see Figure 10).

In summary, Granger causal interactions among EEG networks have larger causal density than their fMRI counterparts. One possible explanation is that more samples were included into the GCA for EEG than for fMRI. Increasing the sample size makes the statistical significance easy to pass the threshold when keeping the SNR [36]. An alternative explanation is that the sensitivity to explore the influence among fMRI ICs decreases rapidly with low temporal resolution, which has been systematically investigated before [31], [37].

## Discussion

In this study, we proposed a novel method to fuse EEG and fMRI in network space where functional network connectivity was explored using Granger causality in each modality, and then the two groups of connectivities are matched using spatial rather than temporal information. This method has the potential to reconstruct the entire network without missing functional networks that are remain undetected by a single modality. Below we discuss its properties and limitation.

### Functional Connectivity

Functional connectivity analysis is particularly valuable for the investigation of coherent activity in distant brain areas. Most previous studies have applied a region-of-interest (ROI) based cross-correlation analysis approach [38]. The networks extracted by this method can also be used in mFNC. However, the result is ROI dependent. In this study, we defined networks by means of spatial ICA, a data-driven approach that is capable of separating independent patterns without prior knowledge about their activity waveforms or locations [6]. As functional networks are spatially independent, they may be temporal correlated, which provides the theoretical basis for the following causality analysis.

Both task- and non-task-related functional networks are extracted by spatial ICA from EEG and fMRI signals. For EEG, as presented in Figure 6, eight FNs were selected for further investigation. Both EEG IC2 and IC7 represent task-related functional networks since they show occipital activities with peaks between 100 and 600 ms after stimulus onset. They may be the result of overseparation from a single spatial pattern, and NESOI does match them with the same network: fMRI IC2. The spatial matching procedure enables mFNC to be robust for the over-separating problem of ICA. EEG IC5 has a negative-going wave with a peak at 200 ms post-stimulus in the central occipital area, which may also reflect a stimulus response. EEG IC1 has a positive activation around the lateral frontal area, which may reflect a later processing. For fMRI, considering the average BOLD signal change, three FNs are positively related to tasks: fMRI IC1, 2 and 3. The amplitudes of the time courses of ICs are larger than ten percent compared to the unit variance (see Figure 7). However, the localization of IC1, 2 and 3 are a little different: IC2 is located in the primary visual cortex and a small area of visual association cortex, while IC1 extends slightly to extrastriate visual cortex. For IC3, activation further extends to bilateral BA19 and a small region in Broca's area (BA44). The fMRI IC9 consisted of multiple areas: the prefrontal, anterior cingulate, and the inferior temporal gyri. This pattern of brain regions is known as the default-mode network (DMN), as described in [39].

### Spatial Matching between EEG and fMRI

In mFNC, fMRI FNs are matched to EEG FNs in the spatial domain using a EEG source inversion method. Spatial matching may be a good alternative for the popularly utilized temporal matching in simultaneous EEG and fMRI recording [16]. Because EEG needs to be down-sampled to the temporal resolution of fMRI, matching in the temporal domain may neglect a large amount of temporal information in EEG signal. Furthermore, as the exact relation between time-courses of BOLD and EEG is complex [40], temporal correlation has the potential risk of producing misleading results. The spatial matching between EEG and fMRI FNs using NESOI leads to a robust and flexible mapping in the common substrate.

In mFNC, there are some modality-specific FNs which are invisible for one modality. This enables our method to access the uncoupled regions implicated in EEG and fMRI signal. The discordance may be associated with the distance between the neuronal population, whose electrical activity generates the EEG signal, and the vascular tree, which provides the blood supply to these neurons [41]. If the electrophysiological activity is transient, it might not induce any detectable metabolic activity changes. In contrast, a number of physiological processes can cause hemodynamic BOLD changes, without EEG correlates. Such example includes neurotransmitter synthesis, glial cell metabolism, or maintenance of the steady-state transmembrane potential. This differential sensitivity to neuronal activity can also arise whenever hemodynamic activity is caused by non-synchronized electrophysiological activity or if the latter has a closed source configuration that is invisible to EEG.

### Exploring Influences between Functional Networks

In general, GCA analysis may be employed for all time series at a unified temporal scale. In this work, the interaction between neuronal populations in mFNC is explored separately by causality analysis within each single modality for two reasons. First, EEG and fMRI data must undergo typical preprocessing in this implementation, including convolution or deconvolution to compensate for the hemodynamic lag before entering the joint data space. Therefore, GCA analysis of all time series ignores the potential bias induced by model mismatches due to variable hemodynamic delays. Second, as we assumed that the matched EEG-fMRI common FNs represent the same neuronal populations, loading both the EEG and fMRI time-courses may lead to an ill conditioned matrix for Granger causality analysis and confusion in interpretation.

There are several potential obstacles in applying Granger causality to fMRI signals. First, BOLD is an indirect measure of neural dynamics and temporal information may be distorted by hemodynamic blurring of the neuronal responses [31]. Second, the hemodynamic response function is known to vary between subjects and such variability has the potential to introduce artifacts when assigning causality [37]. Third, another bottleneck is that the fMRI signals have relatively poor temporal resolution at the order of seconds. However, dynamic interactions between neuronal populations usually take place at a time scale of millisecond. Previous literature indicated that the sensitivity to detect interactions between neuronal populations decreases rapidly with increasing sampling interval (i.e., the whole volume repeat time) [31]. In our method, the spatially matched EEG and fMRI FNs are assumed to represent the same neuronal populations. This assumption allows mFNC to explore the interactions among FNs with different modalities. The autonomous FNs estimated by fMRI GCA may also have information flows when we consult EEG GCA. fMRI network connectivity, in a way, is temporally improved with the help of EEG. For example, there may be an arrow from fMRI IC2 to fMRI IC5 when we consider the EEG information (see the arrow from EEG IC7 to EEG IC6 in Figure 9).

### Multimodal Functional Network Connectivity

Functional segregation (i.e. the brain as an ensemble of functionally segregated areas) and functional integration (i.e. functionally specialized areas are integrated and psychological functions are caused by distributed interactions) are the two main principles of brain function. They both find support with recent developments in functional neuroimaging. As demonstrated in our previous work [18], NESOI is actually a localization method to find functionally segregated neural sources related to a special task. Although localized sources might be sufficient to explain some aspects of pathophysiology, they are not sufficient to fully account for all possible symptoms, clinical course, or treatment considerations, which instead may be more related to the (dys)function of distributed networks [42].

In this work, we attempt to develop an analysis procedure to reveal both functional segregation and functional integration simultaneously. The implicit generative model of mFNC entails a number of assumptions about brain responses. First, brain activity is generated by a set of spatial independent patterns. Each pattern contains multiple regions with temporally coherent activity. Second, these patterns are functionally connected and their temporal dynamics can be modeled as an autoregressive process. Third, EEG and fMRI observe parts of these patterns. In contrast with the models in [43], [44], [45], EEG and fMRI do not need to share a common substrate.

In the real data test, mFNC unveiled the comprehensive relationships among FNs during a visual stimulus task. Because of the retinotopic organization of early visual cortex, stimulation in the left visual field yields the activation in the right occipital cortices. The visually evoked P100 is identified in EEG IC2 and IC7. After source imaging, their locations are mainly overlapped with fMRI IC2, which is located in the primary visual cortex. Then, visual information flows further to the visual association cortex (EEG IC5 and fMRI IC1). EEG IC5 has a negative-going wave that peaks at 200 ms post-stimulus around the central occipital area. As can be seen from the average BOLD signal change at the time of stimulus onset, fMRI IC1 is the task related component, and is partially located in extrastriate visual cortex. A wide variety of visual primitives are processed here [35]. We also find an electrophysiological activation (EEG IC3) in the opposite visual areas. However, this component has no matching fMRI source. Previous studies have identified the DMN as the task-negative network. In mFNC, an edge between DMN and primary visual network is identified and thought to result from the short rest between the simple visual stimulus tasks. In Figure 9, the neuroelectric interaction between EEG IC7 and IC5, and the hemodynamic interaction between fMRI IC2 and IC1, both indicate that there is a bottom-up visual process. The occipital primary visual cortex is the source of visual processing, and its information is flowing to the visual association cortex. Overall, mFNC reveals comprehensive relationships among functional brain networks, which may be helpful to explore the neuroelectric and metabolic responses during checkerboard stimulation. Consequently, we conclude that mFNC provides a unification of different views provided by functional segregation and integration, and may therefore represent an approach that is more akin to actual brain processing.

### Methodological Limitations

We should notice that there are limitations to this approach. As the procedure of mFNC entails a number of different algorithms, any potential problem of them may distort the final result. First, temporal ICA has by far dominated EEG analysis to date. The choice of spatial or temporal ICA should be made according to the characteristics of the underlying signals to be estimated [46]. Spatial ICA of EEG is used here to extract statistically independent spatial components. Although the results reported in the current study are encouraging, one has to note that for single trial EEG data, the ICA assumptions may be inappropriate, and an alternative choice is imposing constraints on the cortex rather than on the scalp [47]. Second, one of the open problems with ICA is how to determine the optimal number of components. Our approach was to contain as many components as the input data, and to implement the ICA several times. The common components across multiple implementations are identified by cluster analysis [20], [48]. However, there are some popular criteria for this issue, such as the minimum description length which has been modified to account for spatial correlation of fMRI data [49]. Third, we employed GCA in event-related single trial data, which implies the assumption that time courses are jointly wide sense stationary. It could be argued that this assumption is inadequate for non-stationarity processes, though our GCA is performed in accordance with many event-related studies [31]. Last but not the least, the generative model of mFNC contains a number of implicit assumptions, which may be obstacles for its application. For example, multiple brain regions may partially overlay in an experiment and the spatial independent assumption is inappropriate in this condition. Our method also may fail if the interactions among neuronal populations are highly nonlinear [50].

### Conclusion

In summary, the novelty of mFNC is that temporal causality is explored separately within EEG and fMRI signals and the common networks between modalities are matched using spatial information. Synthetic data studies demonstrated the potential of mFNC to reveal the correct networks if EEG and fMRI have the same spatial location of neural sources. Analysis of experimental showed that mFNC allows networks to be found that are in accordance with current knowledge of visual processing in the human brain. In addition, mFNC showed some connections that are driven by the high spatial resolution of fMRI and the high temporal resolution of EEG. Compared with the FNC derived from the fMRI modality alone, the presented exploratory analysis showed that mFNC revealed a more complete dynamic picture of the complex brain-state fluctuations underlying cognitive and perceptual processes. A particularly useful application of mFNC would be to examine abnormal relationships among brain networks in psychiatric patients to better understand their neurobiological basis. Combined with single-trial analysis, mFNC might also be used to identify plasticity effects induced by various experimental manipulations. More importantly, this could accelerate the comparative study of the EEG default mode network [51], [52] and the fMRI default mode network [39].

## Supporting Information

Text S1 **Restricted Maximum Likelihood algorithm.**
(DOC)

Figure S1 **Excluded EEG independent components.** For each component, scalp topography (first column), single trial ERP image (second column) and the average EEG (third column) are shown.
(TIF)

Figure S2 **Excluded fMRI independent components.** Sagittal, coronal and axial views of the spatial map are listed for each component. These are scaled to z scores and shown in a maximum intensity projection format. Blue to green represent z values ranging from min to -3.0, and yellow to black represent z values ranging from 3.0 to max.
(TIF)
