# Supplementary material for: Multimodal Functional Network Connectivity: An EEG-fMRI Fusion in Network Space
Source: PLoS One. 2011 Sep 22;6(9):e24642. doi: 10.1371/journal.pone.0024642 (PMC3178514; doi:10.1371/journal.pone.0024642)
Supplement: Text S1 — Restricted Maximum Likelihood algorithm. (DOC) [file pone.0024642.s001.doc]

**Restricted Maximum Likelihood algorithm**

For the topography of each EEG IC, *Y*, we employ an Empirical Bayesian (EB) model [1,2] for its underlying source distribution,

**X** *Φ* **C**_1_)

*Φ*  **C**_2_),

where $Y\in R^{e\times1}$ is one of the EEG topographies with *e* electrodes. **X**$\in R^{e\times d}$ is the known lead-field matrix calculated for the selected head model, and *Φ*$\in R^{d\times1}$ is the unknown distribution of *d* dipoles. $N(\mu,$**C**) denotes a multivariate Gaussian distribution with mean *μ* and covariance **C**. The terms *E*_1_ and *E*_2_ represent random fluctuations in channel and source spaces, respectively. These spatial covariances **C**_1_ and **C**_2_ are mixtures of covariance components at the corresponding levels. At the electrode space level, we assume **C**_1_$=\alpha^{-1}$**I**_e_ to encode the covariance of electrode noise, where **I**_e_ is an *e×e* identity matrix. At the source space level, we express **C**_2_ as the covariance components,

**C**_2_ **V***_i_*,

where is a vector of *k* non-negative hyperparameters that control the relative contribution of each covariance basis matrix, **V***_i_*. The Green function **G**, encodes the neighboring relationships among nodes of the cortical mesh defining the solution space. The *j*th column of the Green function matrix **G** is *G*_j_, encoding neighboring patches weighted by their surface proximity [3].

NESOI employs two different kinds of covariance matrices: **V**_i_^f^ and **V**_i_^e^. **V**_i_^f^ encodes the prior coherence pattern information derived from fMRI [2] and **V**_i_^e^ encodes multiple sparse priors [3] that are sparsely sampled from a subspace of EEG source space that does not contribute to fMRI measurements. To derive **V**_i_^f^, the intensity values in each fMRI IC are scaled to z scores. Voxels with absolute z scores >3 are considered to be activated. Negative z scores indicate that the BOLD signals are modulated oppositely to the IC waveform. A node in the EEG source space is assigned according to the z score of its nearest-neighbor fMRI voxel after spatial registration. All the activated nodes (absolute z scores >3) in each IC show similar temporal dynamics of the BOLD signal, thus we assume they have similar properties for EEG signal generation. The simplest way to construct a covariance component from an IC is to set diagonal terms corresponding to activated nodes to 1, and set the rest to 0. NESOI takes into account the local coherence in source space and introduces the covariance component **V**_i_^f^ as,

**V**_i_^f^ =,

where *W*(i) is a set of activated nodes for *i*th IC, *n*_i_ is the cardinality of *W*(i), and *G*_j_ is the *j*th column of the Green function matrix **G**.

For the remaining source space outside the subspace generated by fMRI IC, multiple sparse priors [3] are employed:

**V**_i_^e^ =,

where $G_{j}$ is evenly sampled from the remaining subspace. In light of its location, this approach can denote right hemisphere components as $G_{j}^{\text{right}}$, and left ones as $G_{j}^{\text{left}}$. Furthermore, homologues are added to form a bilateral component, $G_{j}^{\text{both}}$=$G_{j}^{\text{right}}$+$G_{j}^{\text{left}}$, which models correlated sources in the two hemispheres.

In summary, the spatial priors for NESOI consist of two parts: fMRI IC and multiple sparse priors [3]. The effective number of fMRI ICs is automatically selected using an EB model optimization procedure. After the optimization convergence, the conditional source estimate *Φ* is the Maximum a Posteriori (MAP) estimate, or equivalently, the weighted minimum norm, the Tikhonov solution, and is given by:

*Φ*=*α***C**_2_**X**^T^(*α***X****C**_2_**X**^T^+**I**_n_)^-1^*Y*.

The obtained hyperparameter $\gamma_{i}$ encodes the link between *W*(i) (the *i*^th^ fMRI IC) and *Y* (the topography of an EEG IC). To ensure $\gamma_{i}$ is non-negative, a hyperprior to the hyperparameters is introduced using a log-transform and a Gaussian hyperprior on *Ψ* as *p*(*Ψ*)=*N*(*Г*,**Г**) [4]. In this study we used relatively flat distribution: $p(\psi_{i})=N(-32,256)$. Automatic model selection is used to eliminate redundant parameters during iteration [5]. In the context of our models, this is implemented simply by eliminating the element with expectation smaller than -16. In the termination of algorithm, the posterior distribution of $\psi_{i}$ was Gaussian, and it is considered to be valid prior if its 90% confidence intervals exclude -16.

The generative model is then determined by $\gamma$, and maximizing the model-evidence, *p(Y|$\gamma$)*, is equivalent to maximizing

*Φ*|)*dΦ≈f*

where *f* is the variational “free-energy”, and is equal to

*f* = -*α*(*Y*-**X***Φ*)^T^(*Y*-**X***Φ*)-*Φ*^T^**C**_2_^-1^*Φ*+ln *α*-ln| **C**_2_^-1^|+ln |*α***X**^T^**X**+ **C**_2_^-1^|

-$\frac{1}{2}$(*Ψ*-*Г*)^T^**Г**^-1^(*Ψ*-*Г*)-$\frac{1}{2}$ln |**Г**^-1^|+$\frac{1}{2}$|**Σ**^-1^|+*const*,

where *const* denotes a constant, **Σ** is the conditional covariance of the hyperparameters [4]. *f* can be maximized using a standard variational scheme such as Expectation Maximization (EM) to furnish a tightly bound approximation to the log-evidence [3,6], which also yields sparse matching of the ‘common substrate’ of neuronal activity.

1. Phillips C, Mattout J, Rugg MD, Maquet P, Friston KJ (2005) An empirical Bayesian solution to the source reconstruction problem in EEG. Neuroimage 24: 997-1011.

2. Lei X, Xu P, Luo C, Zhao J, Zhou D, et al. (2011) fMRI Functional Networks for EEG Source Imaging. Human Brain Mapping 32: 1141-1160.

3. Friston K, Harrison L, Daunizeau J, Kiebel S, Phillips C, et al. (2008) Multiple sparse priors for the M/EEG inverse problem. Neuroimage 39: 1104-1120.

4. Friston K, Henson R, Phillips C, Mattout J (2006) Bayesian estimation of evoked and induced responses. Hum Brain Mapp 27: 722-735.

5. Friston K, Mattout J, Trujillo-Barreto N, Ashburner J, Penny W (2007) Variational free energy and the Laplace approximation. Neuroimage 34: 220-234.

6. Lei X, Yang P, Yao D (2009) An empirical bayesian framework for brain-computer interfaces. IEEE Trans Neural Syst Rehabil Eng 17: 521-529.
